# Endogenous ROS levels in *C. elegans* under exogenous stress support revision of oxidative stress theory of life-history tradeoffs

**DOI:** 10.1186/s12862-014-0161-8

**Published:** 2014-07-24

**Authors:** Samson W Smith, Leigh C Latta, Dee R Denver, Suzanne Estes

**Affiliations:** 1 Department of Biology, Portland State University, Portland, 97201, OR, USA; 2 Current address: Department of Biology and Microbiology, South Dakota State University, Brookings, 57007, SD, USA; 3 Biology Department, Reed College, Portland, 97202, OR, USA; 4 Department of Zoology, Oregon State University, Corvallis, 97331, OR, USA

**Keywords:** Aging, Fitness, Free radicals, Lifespan, Resource allocation

## Abstract

**Background:**

The oxidative stress theory of life-history tradeoffs states that oxidative stress caused by damaging free radicals directly underpins tradeoffs between reproduction and longevity by altering the allocation of energetic resources between these tasks. We test this theory by characterizing the effects of exogenous oxidative insult and its interaction with thermal stress and diet quality on a suite of life-history traits and correlations in *Caenorhabditis elegans* nematodes. We also quantify demographic aging rates and endogenous reactive oxygen species (ROS) levels in live animals.

**Results:**

Our findings indicate a tradeoff between investment in reproduction and antioxidant defense (somatic maintenance) consistent with theoretical predictions, but correlations between standard life-history traits yield little evidence that oxidative stress generates strict tradeoffs. Increasing oxidative insult, however, shows a strong tendency to uncouple positive phenotypic correlations and, in particular, to reduce the correlation between reproduction and lifespan. We also found that mild oxidative insult results in lower levels of endogenous ROS accompanied by hormetic changes in lifespan, demographic aging, and reproduction that disappear in combined-stress treatments--consistent with the oxidative stress theory of aging.

**Conclusions:**

Our findings demonstrate that oxidative stress is a direct contributor to life-history trait variation and that traditional tradeoffs are not necessary to invoke oxidative stress as a mediator of relationships between life-history traits, supporting previous calls for revisions to theory.

## Background

Evolutionary life-history theory predicts that tradeoffs; i.e., negative correlations, between fitness-related phenotypes will occur as a result of physiological constraints and/or resource limitations [1]-[4]. Tradeoffs have been widely hypothesized to result from organisms having limited energy stores, the allocation of which will evolve to favor either reproduction or somatic maintenance and longevity [5]-[7]; see [1] for a review. Commonly demonstrated tradeoffs include ‘costs of reproduction’ wherein fecundity is negatively correlated to other traits such as lifespan and susceptibility to oxidative and other forms of stress [2],[8],[9]. Reports of positive or variable correlations between traits where classic theory would predict strong negative correlations [10],[11] have been attributed to the expanded resource budgets typical under laboratory conditions; experimentally limiting resources, it has been suggested, might therefore illuminate true tradeoffs by forcing resource reallocation [11],[12]. Accordingly, negative trait correlations have sometimes been revealed by manipulating food availability and by modulating various stressors [2],[9],[13],[14]; however, others have highlighted weaknesses with the design of such experiments (e.g., introduction of confounding factors such as diet restriction) and argued that resource allocation models may be inadequate to explain observed relationships between life-history traits [15].

Despite such criticisms, a popular idea among evolutionary ecologists has been that oxidative stress caused by damaging free radicals shapes life histories [16] and, through its effects on resource allocation, life-history tradeoffs such as that between reproduction and somatic maintenance—a subject of much recent debate [17]-[21]. Free radicals including reactive oxygen species (ROS) are natural byproducts of aerobic energy metabolism occurring via oxidative phosphorylation at mitochondrial electron transport chains (ETC.) [22]. Organisms have evolved diverse, sophisticated mechanisms to neutralize ROS and protect against or repair oxidative damage; however, mitochondrial ETC. dysfunction and various environmental stressors (e.g., UV and thermal stress) can cause elevated levels of ROS and associated cellular damage [23]-[25] known to accumulate with age [25]-[30]. Oxidative stress results when an imbalance between ROS production and detoxification/repair allows such damage to accrue. Despite their capacity for harm, ROS and other free radicals have vital roles in immune system function, calcium homeostasis, and cell signaling [25],[31],[32]. Organisms must therefore satisfy the requirements of their dependence on mitochondrial ATP production while regulating levels of detrimental ROS byproducts; they must also maintain the beneficial roles associated with ROS while minimizing oxidative damage. In a model where ROS mediate life-history tradeoffs, elevated oxidative damage would shift the allocation of available resources among these tasks. The basic idea is that, when confronted with oxidative stress, organisms can devote energy to reproduction, leaving fewer resources for somatic maintenance (e.g., antioxidant protection or repair of oxidatively damaged biomolecules) and resulting in lasting oxidative damage that directly causes aging and shortens lifespan [3],[16]. Alternatively, resources can be invested into somatic maintenance, enhancing survival and longevity at the expense of investments in other energetically demanding functions like reproduction.

A few key criticisms of this idea have been highlighted [12],[33]-[35]. First, despite the fact that oxidative damage accumulates with age and has been linked to etiology of degenerative human diseases, whether it directly causes senescence and shortens lifespan is still unclear. For example, studies that have modified antioxidant levels generally support the idea that both exogenously and endogenously-acquired antioxidants protect against acute oxidative stress [35],[36], but fail to find consistent effects of these treatments on lifespan in laboratory environments [35],[37]-[41]. Second, maintaining antioxidant protective mechanisms may not be especially energetically costly, although there are exceptions (e.g., mitochondrial uncoupling proteins and glutathione). If organisms do not have to “choose” between devoting resources to reproduction or protective defenses, experiments aiming to force tradeoffs between these expenditures by limiting resources are misguided.

While such findings cast doubt on oxidative stress theories of aging and life-history tradeoffs, most researchers still agree that oxidative stress probably underlies some aspects of aging and may help to determine the span of healthy aging—even if it fails to determine maximal lifespan and related tradeoffs. Consequently, there have been calls for research that shifts the focus from maximal lifespan to other measures of aging [42] while making some account of the interacting factors that determine levels of ROS and associated oxidative damage. Furthermore, it has recently been suggested that experimentally manipulating reproductive effort is the only way to properly test a key prediction of the oxidative stress theory of tradeoffs: that increased reproductive effort is associated with (or directly causes) increased oxidative damage; e.g., [21]. The central argument is that measuring oxidative damage in relation to unmanipulated reproductive effort (especially when resources are not limiting) is unlikely to reveal a substantial cost to reproduction since individuals can alter their reproductive output to balance current with future reproductive efforts. This balancing act is likely influenced by organismal condition such that high-condition individuals better able to acquire resources can both reproduce at high rates and live long, resulting in positive life-history correlations. Unfortunately, experimentally manipulating reproductive output is difficult or impossible in many study organisms (especially in those without parental care) without also introducing undesired side effects. In such organisms, the best available approach may be to manipulate oxidative stress in ways that impose physiological constraints or resource limitations (e.g., access to exogenous antioxidants) while controlling for organismal condition, variation in resource acquisition, and other confounding factors such as food availability. While perhaps less likely to reveal strict tradeoffs than direct manipulations of reproductive effort, such an approach may provide insight into the frequency and extent to which tradeoffs are expected to arise in organisms experiencing endogenous or exogenous oxidative insult.

We investigated the role of ROS in mediating life-history variation and tradeoffs in a single genotype of *C. elegans*, an organism for which tradeoffs appear to be largely absent under standard laboratory conditions [43] but see [44]. We chronically exposed nematodes to different levels of paraquat—an herbicide that generates superoxide anions *in vivo*[45],[46] and measured a suite of life-history traits and rates of mortality and demographic aging. Additionally, we measured ROS levels using a fluorogenic dye-based method that allowed us to approximate the treatment-specific levels of oxidative stress experienced by nematodes [47]. We performed additional assays pairing oxidative challenge with mild thermal or dietary stress, treatments expected to further elevate oxidative stress, to test whether life-history tradeoffs would be further exposed or exhibit context-dependent expression.

## Methods

### Nematode strains and culture conditions

Bristol N2 *Caenorhabditis elegans*, obtained from the Caenorhabditis Genetics Center (University of Minnesota, St. Paul, MN), were used for all experiments. Nematode stocks were stored at −80°C [48] and thawed as needed for all assays. Worms were age-synchronized by standard bleach treatment prior to each assay and grown at either 20°C or 25°C on 60 mm Petri plates containing NGM media with 20 μg/mL streptomycin and OP50-1 *Escherichia coli* as a food source. *C. elegans* were acclimated to their respective temperature and diet regimes (below) for two generations prior to each experiment.

### Paraquat (PQ) treatments

Paraquat (PQ), also methyl viologen dichloride, generates superoxide anions *in vivo*[49]-[52] and is commonly used to experimentally generate exogenous oxidative stress [49]-[51]. NGM plates were supplemented with: 0, 3.23, 32.3 or 64.6 μM PQ in final concentration, hereafter referred to as control, low, medium and high PQ treatment levels, respectively. Nematodes were first exposed to their respective treatments at the egg stage on unseeded plates following age-synchronization. PQ was diluted in sterile M9, which served as the control treatment, and stock solutions were stored at 4°C and made fresh prior to each assay. Two hours prior to plate use, 0.5 mL of the stock PQ solution was added to the surface of each containing 15 mL medium and a lawn of OP50-1 *E. coli*. PQ treatment concentrations were decided upon in preliminary life history assays; PQ levels that dramatically reduced or prevented nematode development to maturity under otherwise standard conditions were avoided.

### Temperature treatments

Phenotypic assays were conducted at either 20°C or 25°C. 20°C is considered the thermal optimum for *C. elegans* based on intrinsic growth rate measurements (e.g., [53]), although this species has broad thermal tolerance and exhibits weak temperature preference [54]. 25°C assays were used to evaluate the effect of mild thermal stress on *C. elegans’* response to oxidative insult. This temperature results in faster generation time and reduced lifespan through processes including a general increase in enzyme activities [55], and reduced reproductive output of *C. elegans* compared to 20°C [10]. The lower reproductive output observed at 25°C is primarily due to a reduced number of functional sperm rather than to differences in oocyte production or egg hatching rates [56]. *C. elegans* is an androdioecious (hermaphrodite/rare male) species in which reproduction under standard laboratory conditions is predominantly sperm limited [56],[57]. Hermaphrodite spermatogenesis is completed during the final larval stage (L4), while oogenesis begins during young adulthood [58],[59]; temperature-induced changes in developmental timing can thus alter the time available for production of one or both types of gametes and form the basis of a tradeoff between generation time and lifetime fecundity [60].

### Diet quality treatments

*C. elegans* were fed a diet of either live or UV-killed OP50-1 *E. coli. C. elegans* cultured on UV-killed *E. coli* experience lifespan extension (e.g., Figure ten in [61]) with no reported cost to reproductive output. This lifespan extension is unlikely to result from dietary restriction [61],[62] or the effects of laboratory adaptation [63]. Rather, worms raised on UV-killed bacteria avoid harmful bacterial proliferation within the gut [61]. However, worms will also lose any potential benefit of feeding on live bacteria; e.g., dietary determinants of lifespan such as coenzyme Q [64]. Thus, despite its benefits for longevity, an UV-killed *E. coli* diet could be considered stressful to *C. elegans* in this regard. Plates for the life history assay using UV-killed bacteria were incubated at 37°C for 4 hours to grow a light bacterial lawn and then irradiated for 2 hours at 76 mJ/cm^2^ in a Logic Class II Type A2 biosafety cabinet (Labconco, Kansas City, MO) containing bulbs producing 253-nm radiation. Plates were stored overnight at room temperature and those with any sign of still-growing OP50-1 colonies were discarded.

### Relative reactive oxygen species (ROS) levels

We measured steady-state ROS in live animals using a method that indicates net oxidant levels and thus reflects the rates of both ROS generation and scavenging by antioxidant systems [65],[66]. We previously found that ROS levels measurement in this way were highly positively correlated (Spearman’s ρ = 0.943, *p* = 0.017) with a survey of oxidative DNA damage (frequency of 8-oxo-dG lesions) conducted on experimentally evolved lines of N2 *C. elegans*[47], suggesting that this method can approximate the level of oxidative stress experienced by an organism. ROS levels were measured for live PQ-treated nematodes raised on live *E.* coli at either 20°C (n ~80 per PQ treatment; N = 316) or 25°C (n ~80 per PQ treatment; N = 319)*.* (The PQ + UV-killed *E. coli* treatments yielded too few and developmentally asynchronous worms for meaningful ROS measurements). Briefly, ROS levels were assessed using confocal imaging of the pharyngeal bulb region of live nematodes labeled with MitoSOX Red (Invitrogen, Carlsbad, CA), a mitochondria-targeted dye that fluoresces when in contact with total mitochondrial oxidants [67]. MitoSOX Red working solutions were prepared as part of the respective PQ treatments and made from a fresh 5 M MitoSOX Red stock in DMSO. A high resolution wide field Core DV system (Applied Precision) equipped with an Olympus IX71 inverted microscope mounted with a Nikon Coolsnap ES2 HQ camera (Advanced Light Microscopy Core Facility, Oregon Health and Science University, Portland, OR) was used to capture images at 60X magnification with a 0.15 second exposure time and 5 μm z-stack widths. ROS levels were acquired by manually enclosing the terminal pharyngeal bulb within each deconvolution-optimized image and obtaining the maximum intensity of the area using ImageJ software (NIH). We applied this procedure to 40 labeled and 10 unlabeled (treatment-specific control) animals for each PQ treatment and calculated final ROS levels as the difference between average maximum pixel intensities (across z-stacks for each individual) for labeled and control worms.

We assessed ROS levels at two carefully chosen time points for nematodes within each treatment group: 34 and 68 hours after L1 stage arrest at 20°C; 24 and 48 hours after L1 stage arrest at 25°C. This timing was chosen based on extensive preliminary study and observed times to reproductive maturation (below) and allowed us to image animals at similar developmental ages; i.e., during larval and reproductively mature stages in each treatment. However, because developmental rates varied among PQ treatment groups, measures of PQ-treated *C. elegans* reflect ROS levels at identical chronological (not developmental) ages. Assaying ROS at exactly identical developmental time points for these worms was not possible since this would necessitate slower-developing worms being exposed to PQ and the florescent label longer than faster-developing worms.

### Life history assays

To determine the independent and combined effects of the PQ, temperature, and diet quality treatments on fitness-related traits, we conducted three separate life-history assays: two using a live *E. coli* food source – one at 20°C (n = 200) and one at 25°C (n = 240) – and a third assay using UV-killed *E. coli* at 20°C (n = 200). For all assays, single L1 stage larvae were transferred from age synchronous populations to individual NGM plates and then transferred daily to fresh plates for at least five days following the first reproductive event to separate them from their progeny. ‘Early reproduction’ was calculated as the number of surviving offspring produced during the first two days of reproduction in the control treatment; ‘late reproduction’ was calculated as the total number of offspring for any remaining days of reproduction (c.f., [68]). Total lifespan was calculated as number of days from L1 arrest until death; survival rates were calculated from the same dataset. Worms were checked daily for survival and data were censored for nematodes that desiccated after crawling onto the side of a plate, which became more frequent in increasingly stressful conditions (Results).

Data from the life history assays were used to calculate relative fitness (*ω*) and age-specific mortality patterns for worms within each assay. Relative fitness was computed for each individual following ([69], eq. 2) as: *ω* = *Σ e*^− *rx*^*l*(*x*) *m*(*x*)*,* where *l(x)* is the number of worms surviving to day *x* and *m(x)* is the fecundity at day *x*, and where *r* is the mean intrinsic population growth rate of the control treatment worms from the appropriate assay. The latter was calculated by solving Euler’s equation, *ω e*^− *rx*^*l*(*x*) *m*(*x*) = 1, for *r*.

For comparative purposes and to further assess whether our experimental treatments differentially affected mortality dynamics, we estimated demographic aging parameters for each PQ treatment and assay. Initial mortality (IMR or “frailty”) and rate of aging (ROA) were estimated by fitting survivorship data to a Gompertz mortality model, which assumes an exponential increase in mortality rate with age [70],[71]. We also report mortality rate doubling time (MRDT) and the median age at which survival = 50% for each assay and treatment as described in [42].

### Maturation rate

Maturation rate; i.e., time to first reproductive event, was assessed for control and PQ-treated worms at each temperature (n ~40 per PQ treatment in each assay; N = 298). Beginning either 50 hours (20°C) or 30 hours (25°C) after L1 stage, nematodes were surveyed hourly for eggs using a dissecting microscope. Start times for these surveys were determined from preliminary experiments and each treatment cohort was removed from the incubator for scoring for identical lengths of time. Once the first eggs were identified, the parental hermaphrodite was sacrificed and the plate incubated overnight to confirm egg viability.

### Statistical analyses

Only relative fitness from the 20°C live *E. coli* assay and late-life reproduction from the 20°C UV-killed *E. coli assay* conformed to normality (Shapiro-Wilk, α = 0.05). Attempts to normalize other datasets through transformation failed for all traits except ROS levels in the 25°C assay. Data for these traits were therefore analyzed both by non-parametric analysis (Wilcoxon/Kruskal–Wallis tests using rank sums) and one-way analyses of variance (ANOVA). When results of these tests agreed, parametric results are reported. Because the three assays could not feasibly be conducted simultaneously, any potential effects of experimental block would not be distinguished. Thus, no formal statistical comparisons are made between assays. We determined whether traits differed among particular pairs of PQ treatment levels within each temperature regime using the Tukey-Kramer HSD test or the non-parametric Wilcoxon method. The above analyses were performed in JMP 9.0.2 (SAS Statistical Inc., Cary, NC). Phenotypic variances, covariances, correlations and bootstrap estimates of their standard errors were generated using H2boot software [72].

## Results

### *In vivo* ROS levels

**Figure 1:**
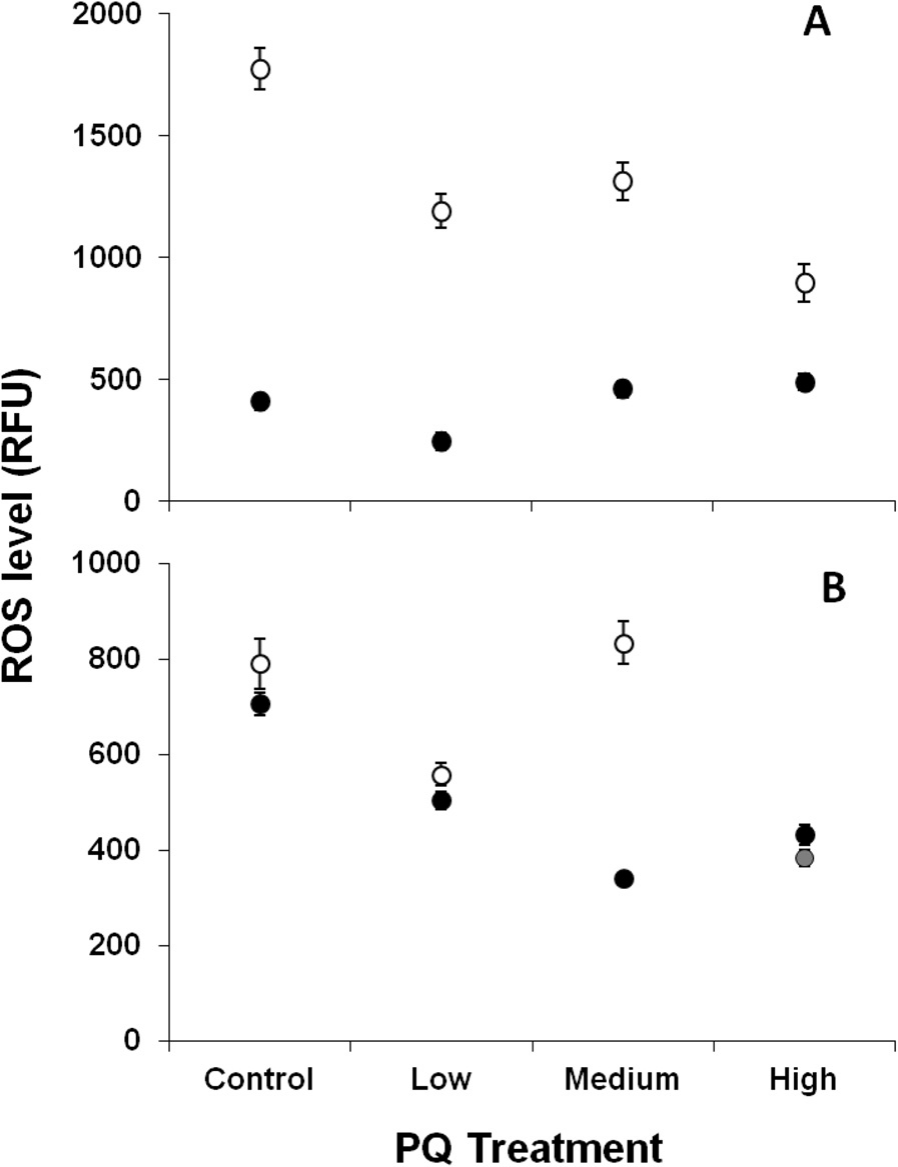
**Relative steady-state ROS levels.** ROS measured in relative fluorescence units of PQ-treated nematodes fed live *E. coli* and raised at either 20°C **(A)** or 25°C **(B)**. Filled symbols show ROS levels measured at earlier, developmentally similar (larval) time points (34 h at 20°C and 24 h at 25°C); open symbols show later, mostly developmentally similar (reproductively mature) time points (68 h at 20°C and 48 h at 25°C) for each assay. Note that the data point for older individuals in the High PQ treatment at 25°C (grey filled symbol) should be interpreted with caution due to the extremely delayed development of this group, and that the change in scale between the y-axes of (A) and (B) is not meaningful (see Results). Bars show one SEM. ANOVA revealed effects of PQ level (F_3, 316_ = 3.320, P = 0.0202), age (F_1_ = 440.1, P < 0.0001), and PQ x age (F_3_ = 21.51, P < 0.0001) on ROS levels measured at 20°C. The same was true for the 25°C assay with significant effects of PQ level (F_3, 319_ = 63.39, P < 0.0001), age (F_1_ = 36.71, P < 0.0001) and PQ x age (F_3_ = 67.33, p < 0.0001) on ROS level.

Because development was severely delayed in higher PQ-treatment groups with UV-killed *E. coli* (below), ROS experiments were restricted to the two live *E. coli* treatments. At 20°C, larva exhibited fairly stable *in vivo* ROS levels with increasing PQ, but had significantly reduced ROS at the low PQ level (Tukey-Kramer HSD, α = 0.05; Figure 1A closed symbols). By contrast, young adult worms showed overall higher ROS levels than larval worms, and a general trend of declining ROS with higher PQ treatment (Figure 1A open symbols). At 25°C, ROS levels of larval worms declined in an approximately linear fashion from control to medium PQ level followed by a significant increase at the highest PQ level (Tukey-Kramer HSD, α = 0.05; Figure 1B closed symbols). Older worms exhibited a variable pattern of ROS level among control, low and medium PQ levels with a sharp decline at the highest PQ level (Figure 1B open symbols). The low ROS level observed for the latter group likely result from their unavoidably delayed development (see Methods). In particular, while control-, low- and medium-PQ-treated worms had reached the young adult stage at the time of these measurements, high-PQ-treated worms were still larvae. Finally, we note that a direct comparison between the 20°C and 25°C ROS experiments (Figure 1A versus B) cannot be made owing to the confocal imaging system filter set and lenses having been changed between studies. However, data within each assay as well as the general patterns of difference between assays can be confidently compared.

### Mortality during life-history assays

Initial sample sizes in each of the four PQ treatments were n = 50 (in the 20°C assays with live and UV-killed *E. coli*) or n = 60 (in the 25°C assay with live *E. coli*). Final sample sizes for each assay and PQ treatment group are provided in Additional file 1: Tables S1-S3. Differences between these numbers reflects data censored for worms that died from desiccation after crawling up the sides of their Petri plates, which became increasingly common in the combined stress treatments and accounted for 54% of the deaths in the highest PQ treatment with UV-killed *E. coli*. Data from these individuals were not considered in any of the analyses that follow.

### Reproductive output

**Figure 2:**
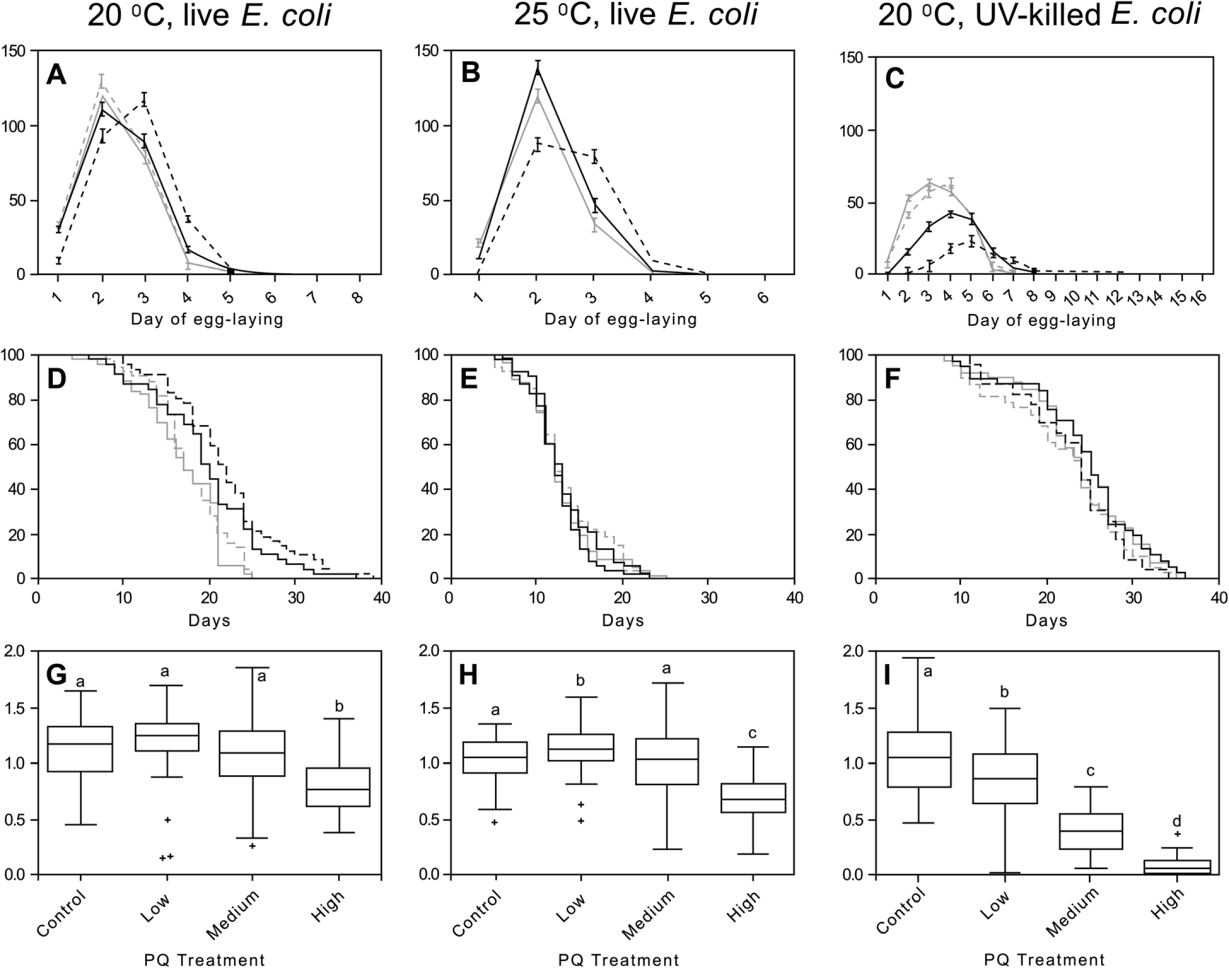
**Summary of phenotypic data for*C. elegans*in each life-history assay.** Assay conditions are shown at the top. **A-C** show daily reproductive schedules for nematodes in each treatment (solid and dashed gray lines = Control and Low PQ treatments, and black solid and dashed lines = Medium and High PQ treatments, respectively). Note the change in x-axis range for panel C. **D-E** show mean survival curves for each treatment. **G-H** report fitness levels for each treatment with horizontal lines = median fitness, boxes = upper and lower quartiles, and error bars = range of values, and letters above each box show groups statistically indistinguishable in post-hoc comparisons. See Additional file 1: Tables S1-S3 for final sample sizes for each assay and PQ treatment group.

In the two assays using live *E. coli*, we observed the expected reduction in reproductive output of control worms raised at 25°C (Additional file 1: Table S2) compared to 20°C (Additional file 1: Table S1), and found that PQ significantly affected average total reproduction (Figure 2A and B).At 20°C, animals experiencing any level of PQ exposure produced on average 12–24 more offspring than untreated controls (Additional file 1: Table S1). Similarly, in worms raised at 25°C, reproductive output was higher for low-PQ (29 more offspring) and medium-PQ (23 more offspring) treatments compared to controls (Additional file 1: Table S2). Within the context of variable diet quality (but constant 20°C rearing temperature), total reproduction was affected by PQ treatment (Figure 2A and C). Diet quality appeared to have little effect on this trait; for instance, there was no significant difference in mean reproduction of control worms fed live *E. coli* (Additional file 1: Table S1) and those fed UV-killed *E. coli* (Additional file 1: Table S3; t = 1.40, P = 0.17), with the caveat that these are from separate experiments. When faced with increasing PQ levels, however, nematodes fed live *E. coli* showed stable or slightly increased reproductive output (Additional file 1: Table S1) while reproduction in those fed UV-killed *E. coli* declined precipitously (Additional file 1: Table S3; Figure 2C). In summary, certain PQ treatment levels (oxidative insult) were associated with minor increases in reproduction, but only in worms fed live *E. coli*.

### Reproductive schedule

In all assays, high PQ level resulted to varying degrees in a delay and extension of the reproductive period (Figure 2A-C). This effect was most pronounced in the assay using UV-killed *E. coli* (Figure 2C). High-PQ treated worms experienced the most severely delayed reproduction, but this delay did not affect mean total reproduction in worms reared on live *E. coli* (above, Additional file 1: Tables S1 and S2) as these animals increased their reproductive output on subsequent days (Figure 2A and B; Additional file 1: Tables S1 and S2). Conversely, higher-PQ treated worms cultured on UV-killed *E. coli* experienced a severe decline in total reproduction, which was not compensated by increased late-life reproduction (Figure 2C and Additional file 1: Table S3).

### Maturation rate

**Figure 3:**
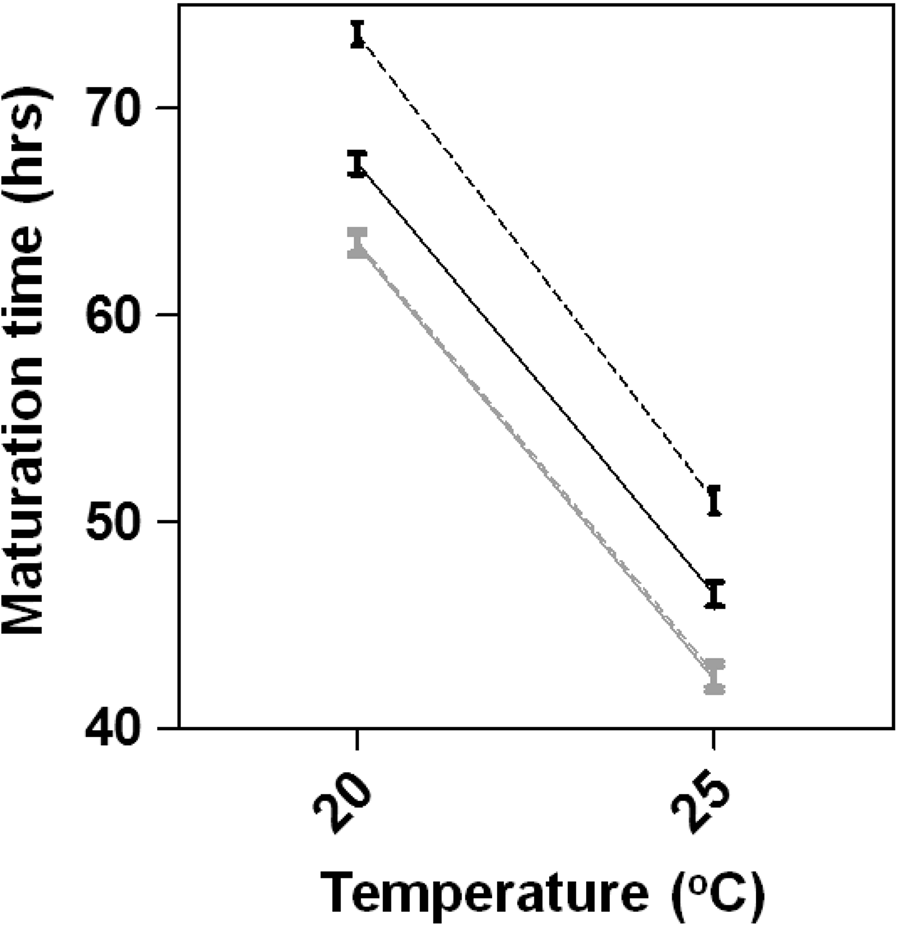
**Maturation rates for*C. elegans*raised at 20°Cor 25°Con live*E. coli*.** Solid and dashed gray (overlapping) lines = Control and Low PQ treatments, and black solid and dashed lines = Medium and High PQ treatments, respectively. Error bars represent one SEM; lines connect equivalent PQ treatments. ANOVA revealed highly significant effects of Temperature (F_1, 298_ = 2695.9, P <0.0001) and PQ level (F_3_ = 110.8, P < 0.0001) but not their interaction (F_3_ = 0.950, P = 0.420) on maturation rate measured as time in hours to first reproductive event.

We measured time to reproductive maturity (Figure 3) to more precisely quantify differences in reproductive timing than in the standard life-history assays. Because time to reproduction was severely delayed in higher PQ-treatment groups with UV-killed *E. coli*, we restricted these assays to the live *E. coli* treatments. The average time to first reproduction in control worms was substantially longer at 20°C as compared to 25°C (mean ± SE; 63.4 ± 4.0 versus 42.4 ± 2.8, respectively) and increasing PQ was associated with further delay in maturation rate. However, the pattern of differences among PQ treatments was strikingly similar at both temperatures; i.e., there were main effects of temperature and PQ on maturation rate, but no temperature-by-PQ interaction (Figure 3 legend).

### Longevity and demographic aging

Compared to nematodes raised under standard laboratory conditions of 20°C with live *E. coli* (Figure 2D), those raised at 25°C showed the expected reductions in maximum lifespan and survival rate (Figure 2E) while those raised on UV-killed *E. coli* showed the expected increase in both traits (Figure 2F). In the 20°C assay with live *E. coli*, nematode survival curves differed significantly among PQ treatments (Log-Rank χ^2^_3_ = 27.40, P < 0.0001) and were accompanied by a large (~11 day) increase in maximum lifespan between the low and medium PQ treatments (Figure 2D). For the other two assays (25°C with live *E. coli* and 20°C with UV-killed *E. coli*), however, mean survival curves were nearly identical among PQ treatments (Figure 2E and F). The same patterns were observed for mean lifespan. Within the 20°C assay, mean lifespan showed a steady increase with PQ level (Additional file 1: Table S1); at 25°C lifespan showed the expected reduction compared the 20°C values, but was also remarkably stable with increasing PQ (Additional file 1: Table S2). Inspection of results from the two assays conducted at 20°C showed that, in contrast to the hormetic effect of PQ on the lifespan of worms fed live *E. coli* at 20°C (Additional file 1: Table S1), worms fed UV-killed bacteria had relatively long lifespans that were stable across PQ treatments (Additional file 1: Table S3). We note that worms that died from bagging (premature hatching of eggs within the parent) were included in the lifespan analyses as is standard in studies of nematode longevity, but these instances did not vary significantly among treatments (12%, 11%, and 14% for 20°C and 25°C + live *E. coli*, and 20°C + UV-killed *E. coli*, respectively) and did not differ from control (no PQ) levels under standard conditions (14% for 20°C + live *E. coli*).

Additional file 1: Table S7 shows demographic aging parameters for each PQ treatment and assay, the patterns of which closely matched those of mean lifespan for this study. The key insights from these results were that aging slows with higher-PQ treatment, but only under standard laboratory conditions (20°C with live *E. coli*). Log-rank tests identified significant differences between survival curves of all pairs of PQ treatment groups in this assay except between the control and low levels and the medium and high levels (data not shown). Both higher temperature and UV-killed *E. coli* conditions masked the effect of PQ on rates of aging: the apparent benefit of PQ for aging disappeared at 25°C where aging was fast and more uniform across PQ levels; conversely, aging was uniformly slow with UV-killed *E. coli* (Additional file 1: Table S7). The same effects are illustrated by comparisons of survival curves for the control populations from each assay, showing that higher temperature significantly increased the rate of aging [Log-rank test for controls from 25°C vs. 20°C assays with live *E. coli*; difference in relative risk (SE) = 0.833 (0.042), P = 0.0003], while an UV-killed bacterial diet reduced the rate of aging [Log-rank test for controls from 20°C with live *E. coli* vs. 20°C with UV-killed *E. coli* assays; difference in relative risk (SE) = 0.787 (0.027), P < 0.0001].

### Relative fitness

Analysis of assays conducted using live *E. coli* detected a strong effect of PQ level but suggested that temperature had little influence on this trait. Although worms cultured at 25°C had reduced total reproduction and lifespan (Additional file 1: Table S2) compared to those cultured at 20°C (Additional file 1: Table S1), their strong early-life reproduction allowed them to achieve similar levels of relative fitness (compare Figure 2H and G). For both the 20°C (Figure 2G) and 25°C (Figure 2H) assays, mean relative fitness was fairly stable with increasing PQ until the highest PQ dose where it declined significantly (Tukey-Kramer HSD, α = 0.05). The magnitude of this decline relative to controls was less extreme at 20°C (Additional file 1: Table S1) than at 25°C (Additional file 1: Table S2), but in both cases was driven by the delayed (but not reduced) reproduction observed at the highest PQ level (Figure 2A and B; Figure 2). Analysis of both 20°C assays also revealed an effect of PQ, and were consistent with an interaction effect of PQ with diet quality on relative fitness. Worms cultured on live *E. coli* (Figure 2H; Additional file 1: Table S1) showed greater resilience to PQ than worms fed UV-killed *E. coli* (Figure 2I; Additional file 1: Table S3). The latter group’s relative fitness declined steadily with increasing PQ, reaching nearly zero at the highest PQ level; this decline resulted from a combination of delayed reproduction (Figure 2C) and lower fecundity (Additional file 1: Table S3).

### Phenotypic variance and life-history trait correlations

Additional file 1: Tables S4-6 show individual trait variances and covariances and correlations between pairs of traits for each assay. Within assays, trait variances had a weak tendency to expand with increasing oxidative stress; however, variances tended to contract slightly under thermal stress (compare Additional file 1: Table S5 to S4).

Visual inspection of Figures 2 and 3 indicates that, at a coarse level, environmental stress may have induced life-history tradeoffs. Compared to worms raised at 20°C under typical lab conditions (Figures 2A,D and 3), those raised at 25°C tended to have slightly increased Day 2 fecundity (Figure 2B), probably due to their accelerated development (Figure 3), but faster aging and reduced longevity (Figure 2E); those raised on UV-killed *E. coli* showed the opposite pattern of tradeoff--far fewer offspring produced early in life (Figure 2C) but slower aging and increased longevity (Figure 2F). These patterns are subtly evident in the proper phenotypic covariances where, for example, relationships between total reproduction and lifespan measured for control worms were positive under normal lab conditions (Additional file 1: Table S4) but significantly lower (Fisher’s z-tests, P < 0.01) and indistinguishable from zero at both 25°C (Additional file 1: Table S6) and in UV-killed *E. coli* conditions (Additional file 1: Table S6). We observed the same pattern within the 20°C live *E. coli* assay where the correlation between these two traits declined monotonically from +0.535 to −0.030 with increasing PQ level (Additional file 1: Table S4). Fisher’s z-tests revealed significant differences between correlations measured in control and high PQ treatment (z = 3.007, P = 0.003) and in low and high PQ treatment (z = 2.055; P = 0.040). Overall, similar patterns held within both assays using live *E. coli* (Additional file 1: Tables S4-S5) where the correlation between reproductive traits became increasingly negative with PQ level--except for high-PQ treated worms at 25°C where early reproduction and lifespan were significantly *positively* associated (Additional file 1: Table S5). The correlation between early- and late-life reproduction also grew increasingly negative at higher PQ levels in the live-*E. coli* assays, but the correlations achieved statistical significance only at 25°C (Additional file 1: Table S5). Although the same patterns did not hold for worms fed UV-killed *E. coli*, a major caveat is that they displayed essentially no variance in early reproduction (Additional file 1: Table S6); correlations involving this trait must therefore be interpreted with caution. In this, the most stressful environment, the magnitude and sign of trait relationships varied across PQ levels with little discernible pattern (Additional file 1: Table S6) but were overall more positive in sign than those measured in the other two assays.

Some of the measured traits (e.g., early and total reproduction) are necessarily positively correlated due to overlapping measurement; the magnitude of these correlations can nonetheless reveal the contributions of early- and late-life reproduction to total reproduction and fitness. In particular, late-life reproduction became more strongly correlated (i.e., contributed more) to total reproduction at higher PQ levels in all assays; the opposite pattern was observed for early-life reproduction (Additional file 1: Tables S4-S6). This was especially true in the 20C assay with UV-killed *E. coli* where late-life reproduction also became significantly positively correlated with relative fitness in the medium and high PQ treatments (Additional file 1: Table S6).

## Discussion

### Nematode life-histories under thermal stress and altered diet quality

Comparing data from control (no PQ) treatments across the three experimental environments yields insight into potential main effects of temperature and diet quality on *C. elegans* life histories. Consistent with previous results (e.g., [10],[56],[73]), worms cultured at 25°C had reduced lifespan, survival rate, maturation time, and total fecundity compared to those cultured at the optimal 20°C. This altered life history did not, however, reveal strict tradeoffs (Additional file 1: Tables S4 and S5). Rather, phenotypic correlations between lifespan and all measured reproductive traits were statistically indistinguishable from zero at 25°C; however, these correlations were uniformly lower than those measured at 20°C. For example, the positive correlation (r_P_ = 0.535) observed between total reproduction and lifespan at 20°C (Additional file 1: Table S4) vanished at 25°C (r_P_ = −0.081; Additional file 1: Table S5), indicating that physiological stress may break positive correlations between traits.

The severe, negative effect of the UV-killed bacterial diet on daily reproductive output of control worms (Figure 2, Additional file 1: Table S3) suggested this was the most stressful of our treatments, but the negative consequences for reproduction were offset by increased mean survival, extended lifespan, and slowed rates of demographic aging (Additional file 1: Table S7), which allowed worms to maintain high relative fitness. Thus, this treatment resulted in tradeoffs in the classical sense; i.e., negative phenotypic correlations between lifespan and both early fecundity and total fitness (Additional file 1: Table S6). This result differs from previous reports that an UV-killed bacterial diet extends *C. elegans* lifespan in a cost-free manner; e.g., [74], but these studies reported only mean lifetime reproduction. We found that diet-stressed worms pay for their extended lifespan with delayed and reduced early reproduction. Even in the absence of PQ, animals in this treatment likely experienced heightened oxidative stress (see Methods). A complication with using these results to directly test the oxidative stress theory of life-history tradeoffs, however, is that the life-history costs and benefits of this diet treatment may have different underlying causes: the life-extending effects of an UV-killed (or growth-arrested) bacterial diet are believed to arise from worms avoiding the harmful effects of bacterial proliferation in the gastrointestinal tract [61], which may or may not reduce levels of oxidative damage, while the costs of this diet for reproduction are likely to result from lost nutrients and/or reduced antioxidant defense [64]. Thus, the above findings cannot tell us whether oxidative stress is at the heart of observed life-history trait relationships, but lead to the preliminary conclusion that environmental stress tends to reduce the strength of the positive correlations between lifespan and reproduction measured in benign conditions--even it fails to reveal hidden tradeoffs.

### Treatment-specific oxidative stress levels

We found that *C. elegans* responded to increasing PQ in both age- and temperature-dependent ways such that 25°C appeared more stressful than 20°C and younger worms were less oxidatively stressed than older worms (Figure 1). During larval stages, nematodes were capable of modulating their endogenous ROS levels under all PQ and temperature treatments, but worms of all stages likely experienced higher levels of oxidative stress in the higher temperature. As previously noted for the 25°C assay, older worms at the highest PQ level exhibited reduced oxidative stress (Figure 1B grey symbol) as a likely consequence of this group's delayed development (Figure 3) meaning that it was comprised of larval worms at the time of measurement. Younger animals’ superior responsiveness to oxidative insult is expected based on their antioxidant capacities: for example, steady-state mRNA levels for the ROS detoxification enzymes glutathione S-transferase and superoxide dismutases were elevated in larval but not adult worms subjected to acute paraquat exposure [75]. Additionally, larval-stage *C. elegans* rely less strongly on metabolic pathways that generate endogenous ROS than do adult-stage worms [76]. Although our data are unavoidably confounded by variation in developmental rates (Figure 3; Methods) and any associated differences in antioxidant capacity or endogenous ROS generation among treatment groups, they shed light on how individuals in the life-history assays experienced the temperature and PQ treatments. Finally, that we failed to obtain sufficient numbers of age-synchronous animals from the UV-killed *E. coli* assay to measure ROS levels is further evidence that *C. elegans* experienced this as the most stressful treatment.

### Nematode life-histories in oxidative- and combined-stress treatments

Chronic PQ exposure yielded slight hormetic effects on life-history traits in dose- and temperature-dependent ways, but only for worms fed live *E. coli*. PQ treatment increased total reproduction at both 20°C and 25°C (Figure 2, Additional file 1: Tables S1-S2). This effect was sustained across all PQ levels at the less stressful 20°C temperature, and was somewhat surprising since self-reproductive capacity in *C. elegans* is predominantly sperm-limited [60] and there was no opportunity for outcrossing in our life-history assays. This result may indicate that the negative effect of PQ treatment on developmental rate (Figure 3) allowed additional time for spermatogenesis to occur by extending the L4 stage (to which spermatogenesis is confined), resulting in higher total reproductive output. Also interesting was that the enhanced reproductive output of worms in the low-PQ treatments--where hormesis was most pronounced at both temperatures--appeared to come at no cost to other traits. Indeed, at 20°C, PQ treatment also had hormetic effects on lifespan and rates of survival and demographic aging--unlike at 25°C where lifespan was short and aging was fast across all PQ levels. The favorable effects of medium- or high-PQ levels on nematode reproduction were, however, always accompanied by delayed development and reproductive timing. Although these worms compensated for lost early reproduction by increasing late-life reproductive output (c.f., [77]), the shifted reproductive schedules severely diminished relative fitness of worms in high-PQ treatments (Figure 2G and H). In light of the fact that ROS levels were significantly lower in low-PQ treated than in control worms at both temperatures (Figure 1), these findings suggest that mild oxidative insult is beneficial for *C. elegans*, resulting in lower endogenous ROS levels and cost-free hormetic effects on reproduction- and, in less stressful environments, aging-related traits. If these lower ROS levels reflect lower levels of oxidative damage as previously observed [47], mild oxidative insult may exert its hormetic effects through enhanced ROS scavenging (and reduced oxidative damage) resulting from the enhanced antioxidant gene expression known to occur in response to PQ [75]. Taken together, the above findings reaffirm that 25°C is the more stressful temperature and indicate that the threshold beyond which oxidative stress becomes costly is reached sooner for worms in combined stress (high temperature + PQ) treatments.

In agreement with the above assertion, all hormetic effects of PQ disappeared when nematodes were raised with an UV-killed bacterial diet (Figure 2; Additional file 1: Tables S3 and S7), our most stressful experimental treatment. Reproductive traits were profoundly negatively affected by PQ treatment in this environment and, despite extended reproductive periods, worms failed to compensate for lost reproductive output later in life. In contrast to the step-wise negative effects of PQ on reproduction in this environment, the UV-killed diet improved survival, extended lifespan, and slowed rates of aging irrespective of PQ level. We speculate that animals in these treatments were pushed to the limits of their capacity to respond to oxidative damage and, in view of the marked decline in relative fitness with increasing PQ (Figure 2I), were aging in unhealthy ways despite being long lived. The more pronounced effects of oxidative insult observed in this environment may be due to reduced availability of exogenous antioxidants. In support of this idea, development of *clk-1* mutants, which are unable to synthesize coenzyme Q (Q_9_), was arrested unless their diet was supplemented with an alternative quinone, Q_8_, from OP50 *E. coli*[78]. In addition to functioning as coenzymes within the mitochondrial ETC., quinones also serve as lipid-soluble free radical sinks. It is not known which other antioxidant molecules *C. elegans* obtains from its diet, but it is reasonable to suppose that only nematodes in the live *E. coli* experiments were acquiring an antioxidant buffer from their living bacterial food source whose own antioxidant systems are known to be upregulated in response to the concentrations of PQ used here [79]-[81] despite the known bacteriostatic effects of this compound [82]. We also acknowledge that these effects may be temperature dependent such that, for example, *E. coli* in the higher temperature treatment were better able to respond to paraquat; further study would be required to identify such interactions if they exist.

Although we were unable to quantify ROS for the long-lived worms in the UV-killed diet treatment, we noted that mean ROS levels in older worms (Figure 1A and B, open symbols) declined alongside rates of aging (RoA; Additional file 1: Table S7) with increasing PQ at 20°C, while both traits were uniformly higher at 25°C. Maximal lifespan (Additional file 1: Tables S1 and S2) showed the opposite trend for both assays. Together with the observation that low PQ had other beneficial effects in the less stressful 20°C environment (above), these findings are consistent with the oxidative stress theory of aging [25], but do not provide a definitive test. In any case, the ROS data show that animals do not experience oxidative stress in direct proportion to exogenous treatment and highlight the benefits of measuring net oxidant levels in live tissue.

Finally, we found no overwhelming tendency for oxidative stress to generate negative correlations between traits measured on the same individuals. Increasing oxidative stress did, however, tend to decouple positive phenotypic correlations and, in particular, to break correlations between lifespan and reproduction—a pattern similar to the one described for control worms between environments. This trend was far more pronounced in the 20°C assay conducted with live *E. coli* (Additional file 1: Table S4) than in the other more stressful treatments where trait correlations became more variable (Additional file 1: Tables S5 and S6). Despite the absence of strict tradeoffs between standard life-history traits, we assert that delayed reproductive timing, which constitutes a major reproductive fitness disadvantage for nematodes (c.f., [83]) observed with increasing PQ levels (Figures 2 and 3) reflects a life-history tradeoff within the framework of somatic maintenance prioritization over reproduction. Because *C. elegans'* response to PQ exposure includes upregulating expression of energetically costly glutathione S-transferase [75], such a tradeoff could be mediated through increased allocation of resources to oxidative stress response at the expense of reproductive fitness. This interpretation makes sense in light of the shared molecular genetic mechanisms of survival and oxidative stress resistance in *C. elegans* such as the insulin and insulin-like growth factor signaling (IIS) and target of rapamycin (TOR) pathways. For instance, mild oxidative and other forms of environmental stress activate the DAF-16/FOXO transcription factor, a master regulator of longevity that is typically repressed by IIS. It is reasonable to believe that our PQ treatments cued the IIS, activating FOXO and signaling the downstream oxidative stress response system [84], leading ultimately to increased survival; e.g., [85]. This suspected tradeoff was more pronounced when nematodes were confronted with an UV-killed bacterial diet and loss of any exogenous antioxidants; here the hypothesized shift toward somatic maintenance (manifested as extreme developmental delay and extended lifespan) came at a major cost to reproductive output and relative fitness. Unfortunately, it was not possible to measure to ROS levels and life-history traits on the same individuals, which would have permitted us to calculate phenotypic correlations between these traits.

## Conclusions

We observed complex and environmentally-dependent effects of oxidative insult on life-history traits that, viewed in light of physiological ROS levels, strongly imply that oxidative stress mediates the relationship between such traits. Criticisms of the oxidative stress theory for life-history tradeoffs are predicated on the absence of tradeoffs *sensu stricto*. Although our study could not avoid all previously criticized aspects of experimental design (e.g., quantifying oxidative stress based on tissue-specific measurement of a single class of oxidants), we nonetheless show that traditional tradeoffs are not necessary to invoke oxidative stress as a mediator of relationships between life-history traits, supporting the call for a revision of this theory [20],[86].

### Ethics

This study does not fall under the approval process by Portland State University's Institutional Animal Care and Use Committee for the ethical use of research animals as it does not involve vertebrate species. This is in keeping with the requirements of the Animal Welfare Act and USDA regulations.

## Competing interests

The authors declare that they have no competing interests.

## Authors’ contributions

SWS and SE designed and executed the experiments. SWS, LCL, and SE analyzed the data. SWS, LCL, DRD, and SE wrote the manuscript. All authors read and approved the final manuscript.

## Additional file

## Supplementary Material

Additional file 1: Table S1. *C. elegans* life-history variation among PQ levels at 20°C on live *E. coli*. **Table S2.***C. elegans* life-history variation among PQ levels at 25°C on live *E. coli*. **Table S3.***C. elegans* life-history variation among PQ levels at 20°C on UV-killed *E. coli*. **Table S4.** Phenotypic variances, covariances, and correlations for *C. elegans* at 20°C on live *E. coli.***Table S5.** Phenotypic variances, covariances, and correlations for *C. elegans* at 25°C on live *E. coli.***Table S6.** Phenotypic variances, covariances, and correlations for *C. elegans* at 20°C on UV-killed *E. coli*. **Table S7.** Treatment-specific estimates of demographic aging parameters. Click here for file
