# Supplementary material for: Endogenous ROS levels in C. elegans under exogenous stress support revision of oxidative stress theory of life-history tradeoffs
Source: BMC Evol Biol. 2014 Jul 24;14:161. doi: 10.1186/s12862-014-0161-8 (PMC4222818; doi:10.1186/s12862-014-0161-8)
Supplement: Additional file 1: Table S1. — C. elegans life-history variation among PQ levels at 20°C on live E. coli. Table S2.C. elegans life-history variation among PQ levels at 25°C on live E. coli. Table S3.C. elegans life-history variation among PQ levels at 20°C on UV-killed E. coli. Table S4. Phenotypic variances, covariances, and correlations for C. elegans at 20°C on live E. coli.Table S5. Phenotypic variances, covariances, and correlations for C. elegans at 25°C on live E. coli.Table S6. Phenotypic variances, covariances, and correlations for C. elegans at 20°C on UV-killed E. coli. Table S7. Treatment-specific estimates of demographic aging parameters. [file s12862-014-0161-8-S1.docx]

Additional file 1

| Table S1. *C. elegans* life-history variation among PQ levels at 20 °C on live *E. coli* | | | | | |
| --- | --- | --- | --- | --- | --- |
| Trait | PQ Treatment | Mean (SEM) | n | F | P |
| Total Reproduction |  |  | |  |  |
| A | Control | 237.32 (7.95) | 50 | χ^2^ = 7.980 | 0.046 |
| B | Low | 258.51 (8.57) | 43 |  |  |
| AB | Medium | 249.58 (8.38) | 45 |  |  |
| AB | High | 260.92 (8.11) | 48 |  |  |
| Early Reproduction |  |  | |  |  |
| A | Control | 151.98 (5.75) | 50 | χ^2^ = 55.03 | <0.0001 |
| B | Low | 163.56 (6.20) | 43 |  |  |
| A | Medium | 141.71 (6.06) | 45 |  |  |
| C | High | 101.52 (5.87) | 48 |  |  |
| Late Reproduction |  |  | |  |  |
| A | Control | 85.34 (5.72) | 50 | χ^2^ = 58.98 | <0.0001 |
| AB | Low | 94.95 (6.17) | 43 |  |  |
| B | Medium | 107.9 (6.03) | 45 |  |  |
| C | High | 159.4 (5.84) | 48 |  |  |
| Lifespan |  |  | |  |  |
| A | Control | 16.82 (0.80) | 50 | 8.371 | <0.0001 |
| AB | Low | 17.79 (0.86) | 43 |  |  |
| B | Medium | 19.71 (0.85) | 45 |  |  |
| B | High | 22.19 (0.82) | 48 |  |  |
| Relative Fitness |  |  | |  |  |
| A | Control | 1.114 (0.04) | 50 | 15.204 | <0.0001 |
| A | Low | 1.195 (0.05) | 43 |  |  |
| A | Medium | 1.080 (0.05) | 45 |  |  |
| B | High | 0.800 (0.04) | 48 |  |  |

Trait means, their standard errors (SEM), and sample sizes (n) are reported for each PQ treatment level. Note Total Reproduction and Early Reproduction were analyzed using Wilcoxon/ Kruskal – Wallis rank sum test with one-way chi-square approximation (see text). Initial sample sizes were n = 50 per PQ treatment; departures from this number reflect missing data from worms that died from desiccation after crawling up the sides of Petri plates. df = 3 for all traits. Letters beneath each trait name show groups statistically indistinguishable in post-hoc comparisons.

| Table S2. *C. elegans* life-history variation among PQ levels at 25 °C on live *E. coli* | | | | | |
| --- | --- | --- | --- | --- | --- |
| Trait | PQ Treatment | Mean (SEM) | n | F | P |
| Total Reproduction |  |  | |  |  |
| A | Control | 176.5 (6.14) | 56 | 5.422 | 0.001 |
| B | Low | 205.7 (6.25) | 54 |  |  |
| AB | Medium | 198.9 (6.31) | 53 |  |  |
| A | High | 178.7 (6.37) | 52 |  |  |
| Early Reproduction |  |  | |  |  |
| A | Control | 143.0 (4.84) | 56 | 41.48 | <0.0001 |
| A | Low | 160.5 (4.93) | 54 |  |  |
| A | Medium | 150.0 (4.98) | 53 |  |  |
| B | High | 88.39 (5.02) | 52 |  |  |
| Late Reproduction |  |  | |  |  |
| A | Control | 34.22 (4.65) | 54 | 26.91 | <0.0001 |
| A | Low | 48.32 (4.83) | 50 |  |  |
| A | Medium | 49.35 (4.74) | 52 |  |  |
| B | High | 91.27 (4.79) | 52 |  |  |
| Lifespan |  |  | |  |  |
|  | Control | 12.71 (0.53) | 56 | 0.491 | 0.689 |
|  | Low | 13.19 (0.54) | 54 |  |  |
|  | Medium | 13.19 (0.55) | 53 |  |  |
|  | High | 12.40 (0.55) | 52 |  |  |
| Relative Fitness |  |  | |  |  |
| A | Control | 1.019 (0.03) | 56 | 39.76 | <0.0001 |
| A | Low | 1.115 (0.03) | 54 |  |  |
| A | Medium | 1.001 (0.03) | 53 |  |  |
| B | High | 0.660 (0.03) | 52 |  |  |

Trait means, their standard errors (SEM), and sample sizes (n) are reported for each PQ treatment level. Initial sample sizes were n = 60 per PQ treatment; departures from this number reflect missing data from worms that died from desiccation after crawling up the sides of Petri plates. df = 3 for all traits. Letters beneath each trait name show groups statistically indistinguishable in post-hoc comparisons.

| Table S3. *C. elegans* life-history variation among PQ levels at 20 °C on UV-killed *E. coli* | | | | | |
| --- | --- | --- | --- | --- | --- |
| Trait | PQ Treatment | Mean (SEM) | n | F | P |
| Total Reproduction |  |  |  |  |  |
| A | Control | 223.9 (7.32) | 39 | 65.78 | <0.0001 |
| A | Low | 209.4 (7.42) | 38 |  |  |
| B | Medium | 148.3 (7.52) | 37 |  |  |
| C | High | 71.04 (9.54) | 23 |  |  |
| Early Reproduction |  |  |  |  |  |
| A | Control | 59.87 (4.37) | 39 | 66.59 | <0.0001 |
| B | Low | 44.84 (3.02) | 38 |  |  |
| C | Medium | 14.97 (3.07) | 37 |  |  |
| D | High | 0.700 (3.89) | 23 |  |  |
| Late Reproduction |  |  |  |  |  |
| A | Control | 164.0 (6.68) | 39 | 30.41 | <0.0001 |
| A | Low | 164.5 (6.76) | 38 |  |  |
| B | Medium | 133.4 (6.85) | 37 |  |  |
| C | High | 70.35 (8.69) | 23 |  |  |
| Lifespan |  |  |  |  |  |
|  | Control | 23.62 (1.09) | 39 | 0.688 | 0.561 |
|  | Low | 22.13 (1.11) | 38 |  |  |
|  | Medium | 24.30 (1.12) | 37 |  |  |
|  | High | 22.91 (1.42) | 23 |  |  |
| Relative Fitness |  |  |  |  |  |
| A | Control | 1.048 (0.04) | 39 | 77.84 | <0.0001 |
| B | Low | 0.848 (0.05) | 38 |  |  |
| C | Medium | 0.389 (0.05) | 37 |  |  |
| D | High | 0.079 (0.06) | 23 |  |  |

Trait means, their standard errors (SEM), and sample sizes (n) are reported for each PQ treatment level. Initial sample sizes were n = 50 per PQ treatment; departures from this number reflect missing data from worms that died from desiccation after crawling up the sides of Petri plates. df = 3 for all traits. Letters beneath each trait name show groups statistically indistinguishable in post-hoc comparisons.

**Table S4.** Phenotypic variances, covariances, and correlations for *C. elegans* at 20 °C on live *E. coli*

| n |  | Total | Early | Late | Lifespan | Fitness |
| --- | --- | --- | --- | --- | --- | --- |
| Control (50) | Total | 2583 (1195) | 1496 | 1088 | 114.7 | 11.66 |
|  | Early | **0.870** | 1486 (476.1) | 10.81 | 66.01 | 10.82 |
|  | Late | **0.724** | 0.009 | 1081 (208.9) | 48.37 | 0.787 |
|  | Lifespan | **0.535** | 0.386 | 0.323 | 21.91 (4.094) | 0.531 |
|  | Fitness | **0.901** | **1** | 0.087 | 0.412 | 0.084 (0.026) |
| Low (43) | Total | 3766 (1441) | 2311 | 1458 | 94.04 | 17.87 |
|  | Early | **0.979** | 1905 (704.2) | 400.3 | 57.61 | 13.57 |
|  | Late | **0.789** | 0.304 | 1060 (192.8) | 36.75 | 4.370 |
|  | Lifespan | **0.394** | 0.338 | 0.275 | 17.78 (3.464) | 0.423 |
|  | Fitness | **1** | **1** | **0.448** | 0.336 | 0.103 (0.036) |
| Medium (45) | Total | 2944 (901.5) | 1691 | 1238 | 63.09 | 14.84 |
|  | Early | **0.768** | 1834 (371.1) | -143.2 | 20.22 | 13.43 |
|  | Late | **0.652** | -0.094 | 1382 (338.5) | 42.57 | 1.413 |
|  | Lifespan | 0.188 | 0.075 | 0.182 | 42.76 (9.178) | 0.123 |
|  | Fitness | **0.880** | **0.987** | 0.120 | 0.059 | 0.109 (0.025) |
| High (48) | Total | 3180 (836.0) | 816.3 | 2359 | -10.55 | 8.532 |
|  | Early | **0.417** | 1298 (218.4) | -479.8 | 18.59 | 8.480 |
|  | Late | **0.821** | -0.257 | 2837 (593.2) | -29.07 | 0.051 |
|  | Lifespan | -0.030 | 0.081 | -0.086 | 43.10 (8.863) | 0.148 |
|  | Fitness | **0.634** | **0.969** | 0.004 | 0.093 | 0.062 (0.012) |

Phenotypic variances appear on the diagonal, covariances above diagonal, and correlations below diagonal. PQ treatment groupings are shown on the left with sample sizes in parentheses. Standard errors for phenotypic variances are shown in parentheses. Correlations statistically different from zero are in bold.

**Table S5.** Phenotypic variances, covariances, and correlations for *C. elegans* at 25 °C on live *E. coli*

| n |  | Total | Early | Late | Lifespan | Fitness |
| --- | --- | --- | --- | --- | --- | --- |
| Control (56) | Total | 1229 (237.1) | 806.9 | 380.0 | -11.17 | 5.381 |
|  | Early | **0.835** | 804.5 (148.3) | -31.40 | 0.383 | 5.281 |
|  | Late | **0.551** | -0.056 | 411.5 (84.75) | -17.95 | -0.042 |
|  | Lifespan | -0.081 | 0.003 | -0.224 | 16.78 (3.894) | -0.028 |
|  | Fitness | **0.808** | **0.979** | -0.011 | -0.036 | 0.038 (0.007) |
| Low (54) | Total | 1880 (472.5) | 909.5 | 839.4 | -28.66 | 6.511 |
|  | Early | **0.811** | 739.0 (183.6) | 128.3 | -15.63 | 4.896 |
|  | Late | **0.759** | 0.185 | 711.5 (158.1) | -42.60 | 1.378 |
|  | Lifespan | -0.155 | -0.135 | **-0.372** | 19.60 (3.344) | -0.155 |
|  | Fitness | **0.819** | **0.980** | 0.274 | -0.188 | 0.037 (0.009) |
| Medium (53) | Total | 2750 (632.2) | 1733 | 1028 | -68.36 | 12.98 |
|  | Early | **0.770** | 1979 (394.6) | -219.1 | -63.77 | 12.64 |
|  | Late | **0.589** | -0.147 | 1243 (388.6) | -12.38 | 0.433 |
|  | Lifespan | **-0.348** | **-0.380** | -0.095 | 15.14 (3.081) | -0.453 |
|  | Fitness | **0.867** | **0.990** | 0.044 | **-0.406** | 0.088 (0.018) |
| High (52) | Total | 2491 (757.8) | 858.1 | 1320 | 31.26 | 7.544 |
|  | Early | **0.442** | 1673 (312.2) | -966.43 | 47.79 | 7.819 |
|  | Late | **0.584** | **-0.510** | 2250 (358.0) | -26.98 | -1.417 |
|  | Lifespan | 0.204 | **0.372** | -0.181 | 10.61 (2.637) | 0.257 |
|  | Fitness | **0.770** | **0.951** | -0.148 | **0.396** | 0.043 (0.011) |

Phenotypic variances appear on the diagonal, covariances above diagonal, and correlations below diagonal. PQ treatment groupings are shown on the left with sample sizes in parentheses. Standard errors for phenotypic variances are shown in parentheses. Correlations statistically different from zero are in bold.

**Table S6**. Phenotypic variances, covariances, and correlations for *C. elegans* at 20 °C on UV-killed *E. coli*

| n |  | Total | Early | Late | Lifespan | Fitness |
| --- | --- | --- | --- | --- | --- | --- |
| Control (39) | Total | 1521 (281.3) | 540.2 | 978.5 | -14.11 | 9.441 |
|  | Early | **0.587** | 593.6 (126.6) | -52.99 | -52.76 | 8.471 |
|  | Late | **0.802** | -0.070 | 1032 (186.3) | 38.79 | 0.964 |
|  | Lifespan | -0.056 | **-0.339** | 0.188 | 44.18 (10.01) | -0.783 |
|  | Fitness | **0.696** | **1** | 0.086 | **-0.341** | 0.129 (0.027) |
| Low (38) | Total | 2204 (958.6) | 436.3 | 1769 | 107.9 | 8.454 |
|  | Early | **0.486** | 434.9 (80.34) | 1.255 | -0.972 | 6.070 |
|  | Late | **1** | 0.002 | 1770 (590.0) | 109.3 | 2.335 |
|  | Lifespan | **0.351** | -0.007 | **0.385** | 50.98 (9.305) | 0.098 |
|  | Fitness | **0.655** | **0.997** | 0.196 | 0.047 | 0.091 (0.022) |
| Medium (37) | Total | 2095 (505.1) | 219.5 | 1883.2 | -42.32 | 6.530 |
|  | Early | **0.381** | 171.4 (34.13) | 48.80 | -9.580 | 2.192 |
|  | Late | **1** | 0.090 | 1832 (453.0) | -33.46 | 4.331 |
|  | Lifespan | -0.143 | -0.113 | -0.121 | 45.52 (10.70) | -0.182 |
|  | Fitness | **0.759** | **0.884** | **0.538** | -0.143 | 0.038 (0.006) |
| High (23) | Total | 2493 (537.3) | 39.07 | 2451 | 158.2 | 3.400 |
|  | Early | 0.208 | 10.61 (9.939) | 28.33 | 7.711 | 0.195 |
|  | Late | **1** | 0.154 | 2424 (539.9) | 150.3 | 3.207 |
|  | Lifespan | **0.551** | 0.346 | **0.532** | 36.31 (9.558) | 0.303 |
|  | Fitness | **0.867** | 0.637 | **0.833** | **0.647** | 0.008 (0.003) |

Phenotypic variances appear on the diagonal, covariances above diagonal, and correlations below diagonal. PQ treatment groupings are shown on the left with sample sizes in parentheses. Standard errors for phenotypic variances are shown in parentheses. Correlations statistically different from zero are in bold.

**Table S7.** Treatment-specific estimates of demographic aging parameters.

| **PQ level** | **IMR (days**^-1^) | **ROA** | **MRDT (days)** | **Median** |
| --- | --- | --- | --- | --- |
| **20 °C, live *E. coli*** | | | | |
| Control | 0.005216 | 0.2217 | 3.12651 | 16 |
| Low | 0.004538 | 0.2061 | 3.36316 | 16 |
| Medium | 0.01263 | 0.1214 | 5.709614 | 19 |
| High | 0.011251 | 0.1053 | 6.582594 | 21 |
| **25 °C, live *E. coli*** | | | | |
| Control | 0.015227 | 0.1908 | 3.632847 | 11 |
| Low | 0.014162 | 0.1781 | 3.891899 | 11 |
| Medium | 0.014997 | 0.1943 | 3.567407 | 12 |
| High | 0.013801 | 0.2271 | 3.052167 | 11 |
| **20 °C, UV-killed *E. coli*** | | | | |
| Control | 0.007965 | 0.1157 | 5.9909 | 23 |
| Low | 0.009262 | 0.1242 | 5.580895 | 23 |
| Medium | 0.010635 | 0.1069 | 6.484071 | 24 |
| High | 0.009503 | 0.1333 | 5.199904 | 23 |

For each assay and PQ treatment, point estimates of initial adult mortality (IMR), rate of aging (ROA), mortality rate doubling time (MRDT) and the median age at which survival = 0.5 (Median) are reported. IMR = e^ intercept of regression of ln ux on age class; RoA = slope of the same regression. MRDT = (ln(2)/RoA), and Median = age at which survival or lx = 0.5. Sample sizes for each treatment are the same as those in Tables S4-S6.
